# Humidity-Induced Structural Transformation in Self-Organized Polymer–Surfactant Multilayer Nanofilms

**DOI:** 10.1021/acs.langmuir.5c04237

**Published:** 2025-10-23

**Authors:** Egor A. Bersenev, Phillip Gutfreund, Valentina Rein, Andrei P. Chumakov, Oleg V. Konovalov, Wuge H. Briscoe

**Affiliations:** † School of Chemistry, 1980 University of Bristol, Cantock’s Close, Bristol BS8 1TS, United Kingdom; ‡ 55553 European Synchrotron Radiation Facility (ESRF), 71 avenue des Martyrs, 38000 Grenoble, France; ¶ Institut Laue-Langevin (ILL), 71 Avenue des Martyrs, 38000 Grenoble, France; § Deutsches Elektronen-Synchrotron, Notkestrasse 85, Hamburg 22607, Germany

## Abstract

We have investigated the humidity-responsive structure of polymer–surfactant multilayer nanofilms composed of a hydrophilic maleic acid polymer and an amphoteric amine oxide surfactant (C12-AO). Neutron reflectometry (NR) revealed the presence of a smooth thin film comprising several surfactant bilayers intercalated with the polymer. Upon exposure to increased humidity, the nanostructured film reorganized into a vertically stratified multilayer structure with polymer chains located in the vicinity of hydrated surfactant headgroups, with further humidity increase leading to the formation of surfactant-rich domains, indicating a high mobility of the surfactant molecules in the polymer matrix. Off-specular neutron reflectometry and grazing-incidence X-ray scattering revealed the presence of surfactant nanocrystals in the as-prepared film, which diffused to form surfactant-rich domains upon exposure to humidity, thus providing a reservoir of surfactants. These findings help elucidate a mechanism of structural transformation in dried polymer–surfactant films, thus suggesting an avenue for engineering an antimicrobial coating with longevity and a humidity-activated release of active molecules using two-dimensional confinement effects.

## Graphical abstract

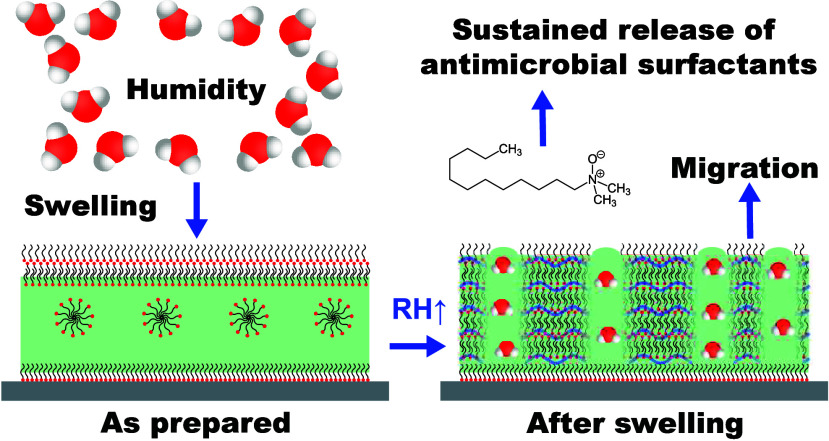

## Introduction

Understanding polymer–surfactant interactions at the interface and in solution is of fundamental importance and practical relevance.^1–6^ Polymer–surfactant complexes have been reported to form a stable ordered mesophase in solution.^7,8^ The formation mechanisms of hybrid polymer–surfactant (P–S) films at the air–liquid interface have also been studied.^1,9–11^ Various surfactants could form complexes with hyperbranched poly­(ethylenimine) (PEI) at the air–water interface,^9,12^ with the electrostatic attraction between the polymer and the surfactant as the driving force. Fine-tuning of such interfacial structures could be achieved by exploiting the pH responsiveness of the polymer, which affects the balance between hydrophobic and electrostatic interactions as well as control over two-dimensional (2D) confinement effects. This approach allows various self-organized structures to be produced for different properties and functionalities by tuning the strength of the driving forces through tailoring the polymer and surfactant architecture.

The multilayer architecture of the films often enhances the functionality of thin films. For instance, polymer–surfactant multilayer films have demonstrated antimicrobial efficacy due to the capacity for surfactant diffusion through the film, sustaining the provision of antimicrobials.^13–15^ Incompatibility between perfluorinated surfactants and charged PEI has also been exploited to produce stratified lamellar films that mediated very low friction coefficients.^16^

Fabrication of polymer–surfactant multilayers at interfaces typically employs multistep preparation protocols with complex procedures, involving dip-coating, vacuum drying, curing, infusion, and incubation.^17–19^ In comparison, the film fabrication route exploiting the spontaneous polymer–surfactant organization is relatively more straightforward. For instance, it has been shown that polymer–surfactant multilayer vesicles in solution may be transferred onto the solid substrate by spin-coating to produce a responsive multilayer coating.^20^

In order to tune the multilayer film structure, it is important to understand the correlation between the structure of self-assembled aggregates in solution and the ensuing interfacial structure, especially for films prepared by spin- or dip-coating from polymer and surfactant mixtures, with several attempts reported previously.^16,20–23^ However, understanding the phase behavior of polymer–surfactant complexes at interfaces remains a challenge due to the templating and confinement effects of the surface, which could induce complex phase behaviors,^24–26^ as well as a rapid transition during the coating process that is difficult to access^27^ and solvent deficiency in the dried film that would lead to the appearance of a phase, different from predicted.

Here, we have studied the correlation between the solution structure of a flexible synthetic maleic acid copolymer complexed with an amphoteric amine oxide surfactant and the structure of the spin-coated films from their complexes using neutron reflectivity (NR) and X-ray scattering. Alkyl amine oxide is readily biodegradable under aerobic and anaerobic conditions,^28^ with high biocompatibility as well as antimicrobial activity,^29,30^ making it a candidate for biodegradable antimicrobial formulations in home and personal care products. Synthetic polymers are widely available and can provide highly stable complexed films, beneficial for coating longevity and mechanical properties. Furthermore, through neutron-contrast variation, reorganization of the spin-coated film in response to a change in humidity could be evaluated, elucidating the surfactant distribution and shedding light on the mechanism of surfactant migration in the polymer matrix.

## Materials and Methods

### Materials

A copolymer with alternating units of methyl vinyl ether and maleic anhydride (Gantrez S-95) with an average *M*_w_ ≃ 216 000 and *M*_n_ ≃ 80 000 g/mol was provided by Procter & Gamble (UK) in the form of a 35 wt % aqueous solution. *N*,*N*-Dimethyldodecylamine *N*-oxide (C12-AO) (Sigma 40234 BioXtra, ≥99%), monoethanolamine (MEA) (Sigma 15014, ≥99%), and deuterated *N*,*N*-dimethyldodecylamine *N*-oxide-*d*_31_ (d-C12-AO) (CortecNet, Paris-Saclay, France) were used as received.

Round Si blocks (2 in. in diameter) with a (100 orientation, polished on one side, were purchased from Siltronix and used for neutron reflectometry. Before spin-coating, wafers were cleaned by being sonicated in pure acetone, Milli-Q water, and ethanol for 5 min in each solvent, dried with a flow of nitrogen, and placed in a laminar flow hood until sample preparation. Square Si wafers with a (100) orientation were purchased from Siltronix and used for X-ray experiments. The preparation procedure was identical.

### Sample Preparation

Stock solutions of the polymer and surfactant were mixed in Eppendorf tubes by shaking at 35 °C for 30 min in a thermal mixer before being stored at room temperature for 2 weeks before further use. Thin films were prepared by spin-coating the solutions at 4000 rpm for 90 s and then immediately transferred to the measurement cell, where they were allowed to equlibrate at 2% relative humidity of D_2_O in order to control the exchange of protons and the consequent change in the scattering length density. The films were subsequently dried in the nitrogen atmosphere for measurement. The spin-coated films consisted of 1 wt % S-95 polymer, 0.5 wt % MEA, and 50 mM (1.15% wt) C12-AO surfactant. The polymer:surfactant ratio in the dried film is assumed to be the same as that in solution used for film preparation.

### Neutron Reflectometry (NR)

The NR measurements were conducted on a time-of-flight horizontal reflectometer (FIGARO) at the Institute Laue-Langevin (ILL, Grenoble, France).^31^ The resolution of momentum transfer *q* was set to 5%. Off-specular scattering was recorded using a 2D neutron detector.

The data were recorded at two incident angles, 0.7° for low *q* and 3° for high *q*, resulting in a *q* range from 0.01 to 0.28 Å^–1^. Low-*q*-range data were collected first for 5 min, followed by the high-*q* measurement, before the repeat acquisition of the low-*q* data to improve statistics and verify the sample stability and integrity. No degradation of the samples was detected during the measurement.

For the measurement, four samples were enclosed in a custom-made chamber, which allowed the relative humidity to be controlled between 0% and 90%, with the temperature remained constant at 25 °C using a thermostatic bath. The humidity was controlled by adjusting the flow rate of a mixture of dry nitrogen and saturated D_2_O vapor using four flow meters, with the humidity level monitored by a humidity sensor placed inside the cell in the proximity of the sample. NR experiments under dry conditions were performed by flooding the sample chamber with dry nitrogen. After temperature stabilization, the sample was allowed to equilibrate to the new humidity levels for at least 15 min before starting the acquisition of the reflectivity curves. The obtained data set is available at the ILL repository.^32^

### Grazing-Incidence Diffraction (GID)

The GID measurements were conducted on beamline ID10 at the ESRF in Grenoble, France. The experiments were conducted using a monochromatic X-ray beam with an energy of 22 keV, corresponding to a wavelength λ of 0.056 nm. The diffracted intensity was recorded with a Mythen2 linear photon-counting detector (Si sensor, 450 μm thick) positioned 430 mm behind the sample. A Soller collimating slit with a resolution of 0.08° was placed before the detector to reduce the parasitic scattering signal. The obtained data set is available at the ESRF repository.^61^

### Grazing-Incidence Small Angle X-ray Scattering (GISAXS)

GISAXS measurements were carried out on beamline P03 at the PETRA III storage ring at DESY (Hamburg, Germany). The experiments were conducted in the grazing-incidence geometry using a photon energy of 11.87 keV, corresponding to a wavelength λ of 0.105 nm, with a pixel array detector (Pilatus3 2M, Dectris Ltd., Switzerland) at a 5200 mm sample–detector distance. Both direct and specular reflected beams were covered with a circular beamstop to avoid detector saturation. The grazing angle was set to 0.4°, which is greater than the critical angle of the Si substrate at this energy (α_c_ = 0.151°) in order to separate the Yoneda band from the direct beam and specular reflection. Resulting 2D images were converted into the data in the *q* space and analyzed using BornAgain code^34^ and custom-made Python scripts.

### Small Angle X-ray Scattering (SAXS)

SAXS measurements were carried out on beamline ID02 at ESRF in Grenoble, France.^35^ The experiments were conducted in the transmission geometry using a photon energy of 12.23 keV, corresponding to a wavelength λ of 0.101 nm. Measurements were performed with an Eiger2 4M pixel array detector (Dectris Ltd., Switzerland), at 0.8 and 10 m sample–detector distances, covering a *q* range of 0.006–10 nm^–1^. Static SAXS measurements were carried out in a flow-through capillary setup, with a capillary diameter of 2 mm, in combination with a 28-port automated sample changer, with transmission measured simultaneously. Radiation damage was checked by making a series of test exposures, and only the data that showed no change during the exposure were further analyzed. The recorded 2D data were normalized to an absolute intensity scale before being regrouped to obtain the one-dimensional (1D) SAXS profiles. The corresponding background was then subtracted from each of the 1D profiles. The obtained data set is available at the ESRF repository.^36^

## Results and Discussion

### Out-of-Plane Structure of Polymer–Surfactant Complex Films

To determine the distribution of the surfactant in the spin-coated film perpendicular to the interface, two isotropic contrasts were investigated: a polymer with h-surfactant (C12-AO), swollen with D_2_O vapors, and a polymer with d-surfactant (d-C12-AO), swollen with D_2_O vapors. Visually, the spin-coated film was smooth and highly sensitive to the relative humidity, which was evident from its change in color from light yellow to deep blue with an increase in humidity.

The presence of the intensity oscillations in the reflectivity curve, known as Kiessig fringes,^37–40^ in the low-*q* (*q* < 0.1 Å^–1^) region in the NR profiles in dry N_2_ and under 27%, 55%, and 80% relative humidity (RH) and after drying (Figure 1) is consistent with a film with a well-defined thickness, with a broad Bragg peak also present at *q* ≃ 0.18 Å^–1^ (Figure 1A, indicated by small arrows), indicating the periodicity in the structure in the as-prepared film placed into the dry nitrogen atmosphere (0% RH). For the films with d-surfactants (Figure 1B), the critical edge in the NR profile was shifted to a higher *q* value (*q*_c_ ≃ 0.017 Å^–1^ compared to *q*_c_ ≃ 0.012 Å^–1^) for the h-surfactant films, indicating a higher average scattering length density (SLD), while the Bragg peak was less pronounced.

**Figure 1:**
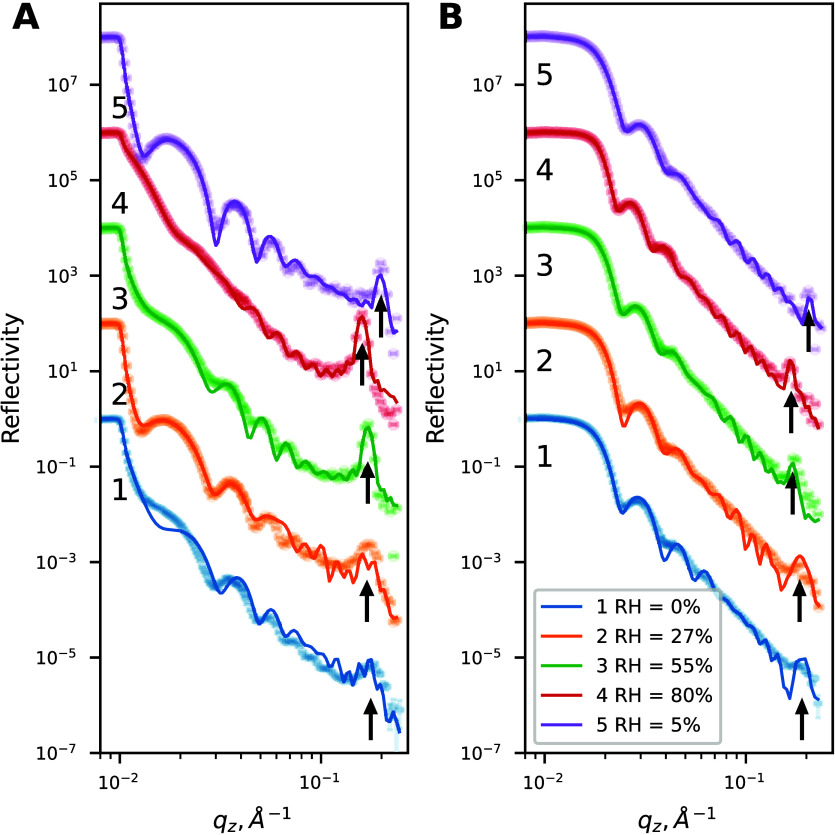
Neutron reflectometry profiles for films containing (A) h-surfactant and (B) d-surfactant. Thin solid lines represent fits to the data, as described in the text. The arrows indicate the Bragg peak for each curve. NR curves 1–5 are scaled by 2 orders of magnitude sequentially for the sake of clarity.

With an increase in RH (Figure 1), the Bragg peak shifted from *q* = 0.175 ± 0.001 Å^–1^ (with a corresponding *d*-spacing of *d*_B_ = 3.55 nm) in dry N_2_ to lower *q* values, as listed in Table S1, indicating an increase in the multilayer *d*-spacing and swelling of the film. This was accompanied by the decrease in the width of the Bragg peak, indicating an increase in the average size of the coherently scattering domain; i.e., the number of ordered layers in the multilayer increased. Concurrently, the Bragg peak intensity increased with RH for both contrasts, although to a lesser extent for the films containing d-surfactants.

In comparison to the lamellar spacing of the pure C12-AO surfactant film (cf. Supporting Information S3) *d*_B_ = 3.2 nm, that of the mixed film is slightly higher (*d*_B_ = 3.55 nm) for the as-prepared mixed film, consistent with the inclusion of polymer moieties between the surfactant headgroups.

Fitting measured reflectivity in the low-*q* region (*q* ≤ 0.1 Å^–1^) (Figure S1) to a single-layer model yielded thickness *d*_tot_ and volume fraction of the solvent ϕ_D_2_O_ in the film, assuming a uniform distribution of the polymer, surfactant, and solvent. Furthermore, fitting the Bragg peak to the Gaussian profile gave center position *q*_B_ and width *w*_B_. These values were used to calculate the *d*-spacing as *d*_B_ = 2π/*q*_B_ and the coherence length perpendicular to the substrate as τ = 2π/(2.355*w*_B_), where 2.355 is the full width at half-maximum (fwhm). The maximum number of layers was calculated as $N_{L}^{max}=d_{tot}/d_{B}$ . These parameters of the film are plotted in Figure 2 and summarized in Table S1.

**Figure 2:**
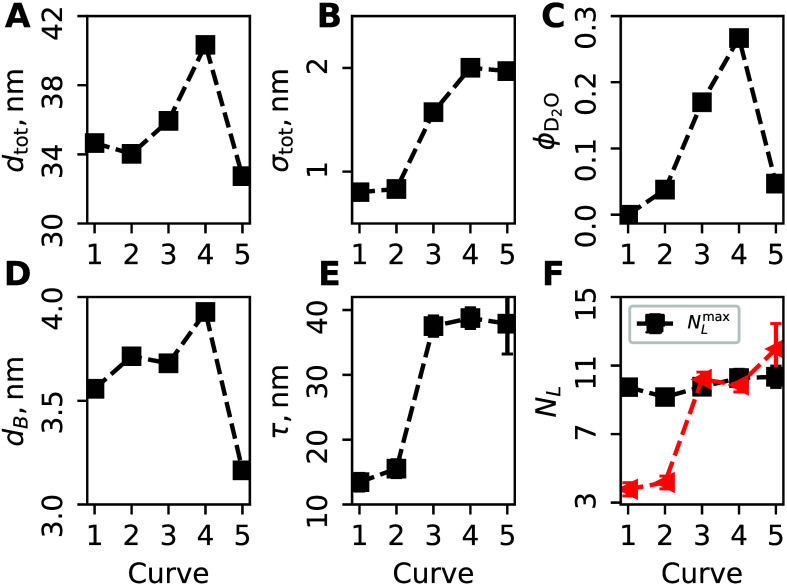
Macroscopic parameters of the film. (A) Total thickness of the film, *d*_tot_, determined by fitting the low-*q* part of the reflectivity curve. (B) Total roughness of the film σ_tot_. (C) Volume fraction of solvent ϕ_D_2_O_. (D) *d*-spacing *d*_B_, determined form Bragg peak fitting. (E) Coherence length τ, determined from the width of the Bragg peak. (F) Maximum number of bilayers $N_{L}^{max}$ (black squares) and numbers of ordered layers at coherent length *N*_L_ (red triangles).

First, the Bragg peak analysis gives the *d*-spacing under dry N_2_ as *q* = 0.17 Å^–1^ with a coherence length τ = 13.4 ± 1.4 nm, corresponding to *N*_L_ = 3.8 ± 0.4 ≃ 4 ordered bilayers, whereas the total film thickness *d*_tot_ = 34.66 nm would accommodate up to 10 bilayers, as indicated by $N_{L}^{max}=9.7\pm 0.2$ . Upon exposure to humidity, the Bragg peak width decreased, indicating an increase in the coherence length. As indicated in Figure 2F, the maximum number of bilayers in the film remained almost constant, even though the total film thickness changed significantly, with an increase in $\frac{d_{tot}^{RH=80}-d_{tot}^{RH=0}}{d_{tot}^{RH=0}}=16.4\%$ , corresponding to the difference between the “as-prepared” film and swollen at 80% RH. Furthermore, exposure to humidity led to a reversible change in the total film thickness *d*_tot_ with a higher roughness σ_tot_ as shown in panels A and B of Figure 2.

These parameters of the nanofilm allow us to construct a more detailed model of the film structure, depicted schematically in Figure 9. First, the periodic bilayer structure was modeled as a four-slab structure, with the inner slabs representing the hydrophobic region consisting of surfactant tails and no solvent and the outer slabs representing the polymer moieties, water, and surfactant headgroups. To account for the inhomogeneity of the film, reflectivity was calculated as an incoherent sum from the structures containing *N* and *N* + 1 repeat units. Only the thickness and solvent volume fraction ϕ_D_2_O_ of the two slabs were allowed to vary within small bounds, based on the molecular dimensions. The incoherent sum means that the reflectivities from two structures were calculated separately and summed with their respective weights, summing to unity. This procedure was chosen to account for film inhomogeneities, as the coherence length of the neutron beam is much smaller than the footprint of the beam on the sample.

Furthermore, the reflected intensity at high humidity was calculated as an incoherent sum of the reflectivities from two different structures: one structure containing a multilayer and another described as a uniform film with one wetting layer of the surfactant near the substrate. The coverage of each component of the film was allowed to vary so that the scaling coefficients would sum to unity. Models containing only multilayer structures did not produce good fits to the specular reflectivity profile at high humidity. Subsequently, a fitted model was reconstructed to extract the volume fraction profiles of the film components, as shown in Figure 3A for the multilayer structure and Figure 3B for the uniform layer at 80% RH.

**Figure 3:**
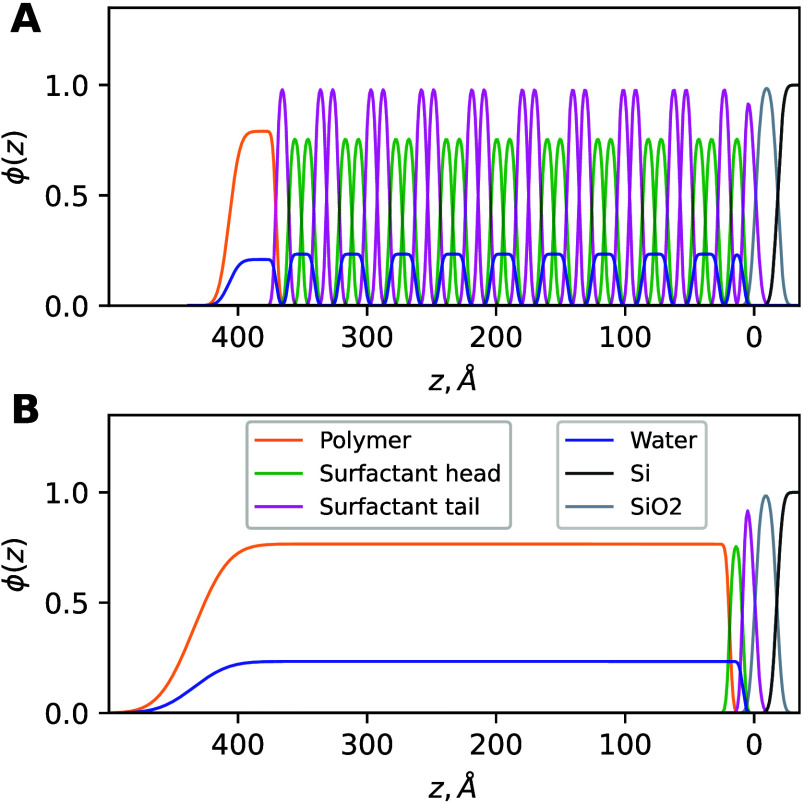
Volume fraction distribution of the components in (A) the multilayer part of the film and (B) the uniform part of the film. The dark gray line represents the Si substrate. The light gray lines represents the SiO_2_ layer. The magenta lines represent the hydrophobic domains. The green lines represent the hydrophilic domains. The orange line represents the polymer layer. The blue line corresponds to the solvent volume fraction.

The volume fraction ϕ­(*z*) profile in Figure 3A shows that the periodic part of the film contains nine repeat bilayers and one surfactant monolayer at the substrate with a thickness that is allowed to vary independently. Figure 3B shows the volume fraction distribution of the components in the film. The difference in the total film thickness between the periodic (Figure 3A) and uniform (Figure 3B) parts is about 35 Å, which is partially compensated by the increase in the total roughness of the uniform part.

Furthermore, at 55% and 80% RH, a thin uniform layer of the polymer was placed atop the multilayer structure to account for the presence of a non-stratified capping layer. This is explained by the high hydrophilicity of the polymer. For each humidity, the water content (ϕ_D_2_O_) in the hydrophilic layers was allowed to vary between 0.05 and 0.9.

The resulting fits are shown in Figure 1. While the model was highly constrained, the main features of the reflectivity curves, such as the position of the critical angle, the Kiessig fringes, and the Bragg peak position, width, and intensity, are reproduced. It should be noted that some features from the model were not observed in the data. This is attributed to the inhomogeneities in the film over a relatively large area illuminated by the neutron beam (ca. 20 cm^2^), which are not taken into account in the model. Furthermore, the difference between the model and experimental data for the “as-prepared” film containing h-C12-AO is attributed to the incomplete exchange of the labile protons in the film, which can slightly distort the apparent SLD of the film. It should be noted that all of the models in this work do not enforce constraints on molecular volumes of the molecules on each interface; i.e., mixing between the components in layers is unrestricted.

### Lateral Organization of the Film

The layered structure of the film is further confirmed by the presence of off-specular scattering, contributing to the appearance of a Bragg sheet at an angle to the vertical specular reflectivity in the 2D scattering pattern as shown in Figure 4A, indicating the presence of an in-plane periodicity or a so-called roughness correlation.^41,42^ This feature is most pronounced for the h-contrast film at the greatest swelling in D_2_O at 80% RH.

**Figure 4:**
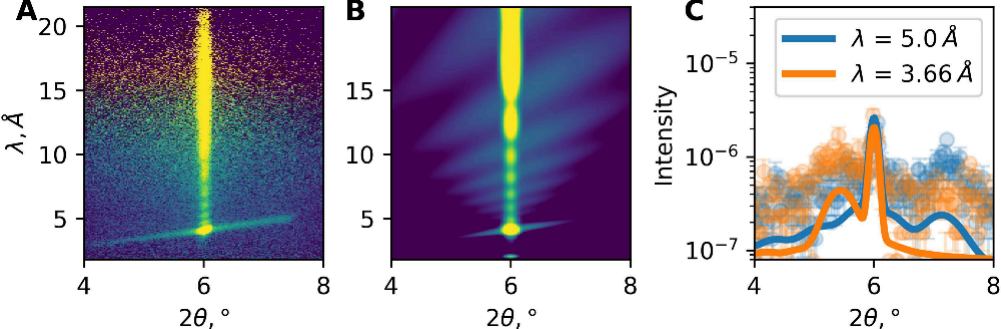
Off-specular neutron reflectivity at high *q* values (θ_i_ = 3° and 80% RH). (A) h-contrast experimental data showing a Bragg sheet at an angle to the vertical specular reflection. (B) Simulated off-specular scattering. (C) Corresponding line profiles at λ values of 5 and 3.5 Å.

Using the distorted wave Born approximation (DWBA) and assuming the conformal roughness of multilayers, it is possible to calculate the off-specular scattering signal.^43,44^ In-plane correlations due to interfacial capillary waves and thermally excited bending may be characterized by two length scales, ξ_1_ and ξ_2_, respectively.^45^ Assuming that the fluctuation amplitude is small, ξ_1_ and ξ_2_ can be decoupled so that height–height (i.e., the vertical displacement from the average interfacial plane) correlation function *u*(*q*)^46^ can be approximated as shown in eq 1:

1 $$\left\langle {\left|u\left(Q_{∥}\right)\right|}^{2}\right\rangle =\frac{\left\langle {\left|u\left(0\right)\right|}^{2}\right\rangle }{1+{\left(Q_{∥}\xi_{1}\right)}^{2}+{\left(Q_{∥}\xi_{2}\right)}^{4}}$$

where *Q*_∥_^2^ = *q_x_* ^2^ + *q_y_* ^2^. Here, $\xi_{1}\propto \sqrt{\gamma /K}$ , where γ is the interfacial tension of the film–air interface and contact *K* (J m^–4^) is the second derivative of the quadratic film–substrate interaction potential, whereas $\xi_{2}\propto \sqrt[{4}]{\kappa /K}$ , with κ being the line bending modulus of the film (in joules). A more detailed explanation of these parameters can be found elsewhere.^47^

Physically, the larger the ξ_1_, the larger the wavelength of the capillary waves, and the larger the ξ_2_, the higher the energy required for the bending of a layer. Analysis of the Bragg sheet scattering allowed the determination of ξ_1_ = 150 ± 30 nm and ξ_2_ = 50 ± 10 nm, giving the ratio $\frac{\gamma }{\kappa }=3.6\times 10^{-3}$ for the bilayers. Assuming the surface tension between the hydrated film and the Si wafer γ_FS_ = 40 mN/m, it yields a bending modulus κ = 22 ± 4 kT.

The calculations, as shown in Figure 4C, show a good qualitative agreement between the measured (circles) and calculated intensity (solid lines), with the simulated 2D image shown in Figure 4B. The intensity of the simulated Bragg sheet appears lower than the experimental intensity, attributed to the underestimated roughness in the model. However, the inclusion of a higher roughness between the layers would lead to a subsequent increase in the scattering length density contrast between the layers to unphysical values. While the roughness value is important for the overall intensity of the Bragg sheet, the Bragg sheet shape is determined by ξ_1_ and ξ_2_, which is in good agreement with the experiment.

Off-specular scattering at low *q* is shown in Figure 5A (h-contrast) and Figure 5D (d-contrast). The presence of the Yoneda scattering at *q* < *q*_c_ indicates inhomogeneities in the film.

**Figure 5:**
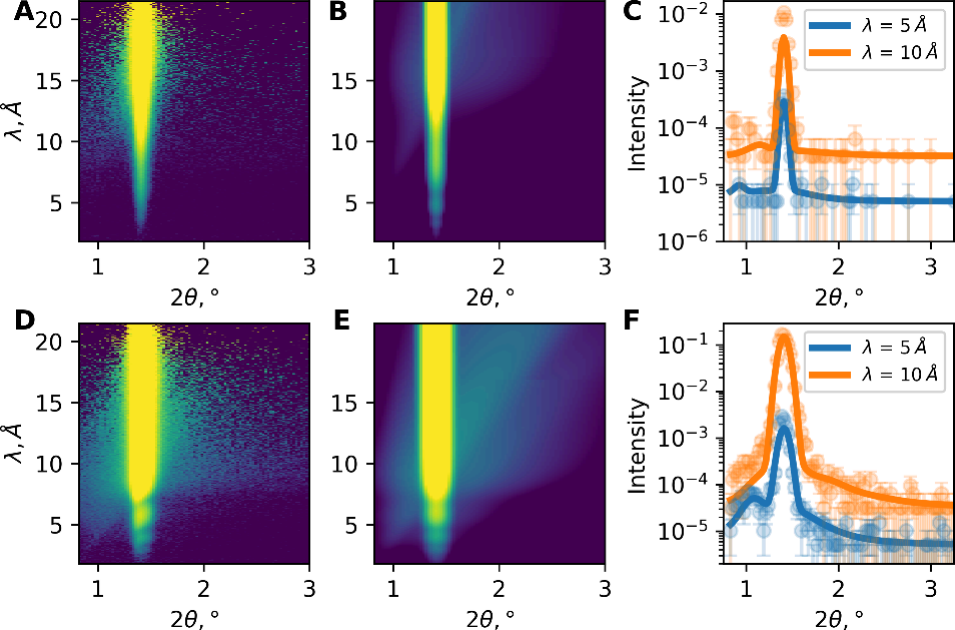
Off-specular neutron reflectivity at low *q* (θ_i_ = 0.7° and 80% RH): (A) h-contrast, experiment; (B) h-contrast, simulated image; (C) h-contrast, line profiles at λ values of 5 and 10 Å (solid lines are calculated fits according to the model, described in the text); (D) d-contrast, experiment; (E) d-contrast, simulated image; and (F) d-contrast, cuts at λ values of 5 and 10 Å (solid lines are calculated fits according to the model, described in the text).

Yoneda scattering was calculated using the DWBA approximation (simulated images shown in panels B and E of Figure 5), and a comparison of the line profiles at different wavelengths with the experiment is shown in panels C and F of Figure 5. The model for calculation was based on a single-layer film of uniform density with shallow cylindrical pores on the film surface with the cylindrical axis perpendicular to the surface, as depicted in Figure 6C. The fitted inhomogeneity radius is *R* = 900 nm, and the depth is *c* = 2.5 nm, with the simulated images shown in panels B and E of Figure 5.

**Figure 6:**
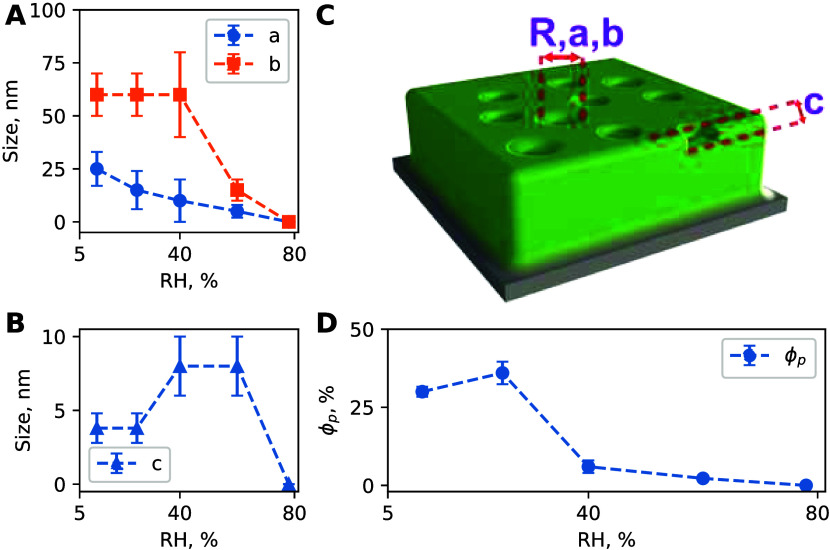
Simulation parameters of GISAXS images. (A) Lateral dimensions *a* and *b* of the ellipsoidal pores. (B) Depth *c* of the ellipsoidal pores. (C) Schematic depiction of the porous surface model used for GISAXS and off-specular NR modeling. (D) Share of the surface, covered in pores ϕ_p_.

The GISAXS experiment confirmed the presence of shallow polydisperse mesopores located near the film surface, similar to the results from the NR off-specular data. The scattering was modeled with a 40 nm film of uniform density with polydisperse semi-ellipsoid shallow pores with an average semi-axis *a* = 22 ± 4 nm and *b* = 60 nm, a polydispersity in all dimensions ϵ = 1.2, and an average depth *c* = 2.3 nm, using the BornAgain code.^34^ Details of the simulation are given in section S7 of the Supporting Information.

As shown in panels A and B of Figure 6, an increase in the relative humidity results in a decrease in mean pore size and depth. Figure 6D shows surface fraction ϕ_p_ of the pores, which also decreases with an increase in the relative humidity. Schematically, the porous structure of the film is shown in Figure 6C. This model was used for both GISAXS and off-specular NR modeling; however, the origin of the scattering is different. The GISAXS signal is due to the presence of shallow mesopores in the film surface. In off-specular NR, Yoneda scattering appears due to the presence of two structures, shown in Figure 3: periodic and uniform. The difference in thickness between these two structures is the origin of such scattering, as the surface of the film is not homogeneous.

The Bragg peaks in the grazing-incidence X-ray diffraction (GIXD) data (Figure 7) revealed the presence of surfactant nanocrystals dispersed in the polymer matrix in the as-prepared film. Pure surfactants formed a film with highly ordered crystals, with a large lateral size, which is evident from the intense and narrow Bragg peaks. In the as-prepared P–S film, similar Bragg peaks were detected; however, the crystals were much less ordered, as indicated by the increased width and decreased intensity of the Bragg peaks. The presence of the Bragg rods in the GIXD profile for the as-prepared P–S film indicates that the surfactant crystals were well-aligned with respect to the substrate. The crystal thickness can be estimated from the width of the Bragg peak in the *q*_*z*_ direction. For our analysis, we chose the most intense Bragg peak at *q*_*xy*_ = 1.43 Å^–1^ and *q*_*z*_ ≃ 0.55 Å^–1^. For the pure surfactant film, the size of coherently scattering domains in the *q*_*z*_ direction was found to be τ_c_ = 12.6 nm; for the as-prepared P–S film, τ_c_ = 10.7 nm. With an increase in RH to 60%, the crystals “melt”, as indicated by the disappearance of the Bragg peaks, with the surfactants uniformly distributed in the polymer matrix. Full reciprocal space maps and details of analysis can be found in section S5.

**Figure 7:**
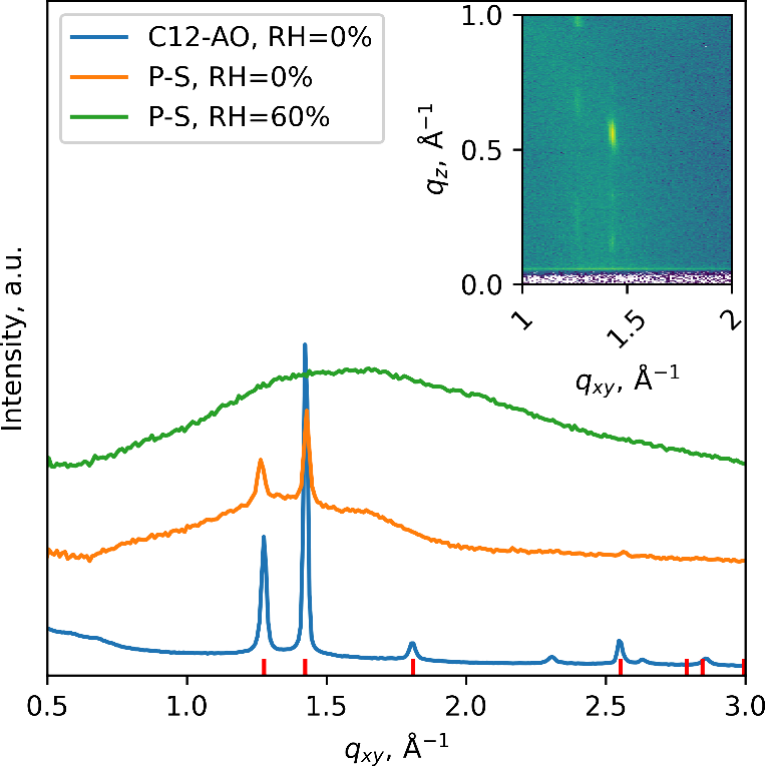
Grazing-incidence X-ray diffraction (GIXD) profile of the pure surfactant film, as-prepared P–S nanofilm, and P–S nanofilm, exposed to high humidity. The inset shows a reciprocal space for the as-prepared P–S film. Red ticks indicate positions of the distorted hexagonal cell of the C12-AO surfactant.

### Structure of Solution Aggregates

SAXS measurements unveiled the solution structure of the polymer–surfactant complexes with the results presented in Figure 8. A typical scattering pattern of a polyelectrolyte solution was observed, with a well-defined correlation peak (with its position depending on the polyelectrolyte concentration) and a power-law low-*q* scattering profile, which can be modeled as a Gaussian chain with an excluded volume and a finite cylindrical cross section.^48^ Interpolymer interactions were described using a PRISM-derived structure factor.^49^

**Figure 8:**
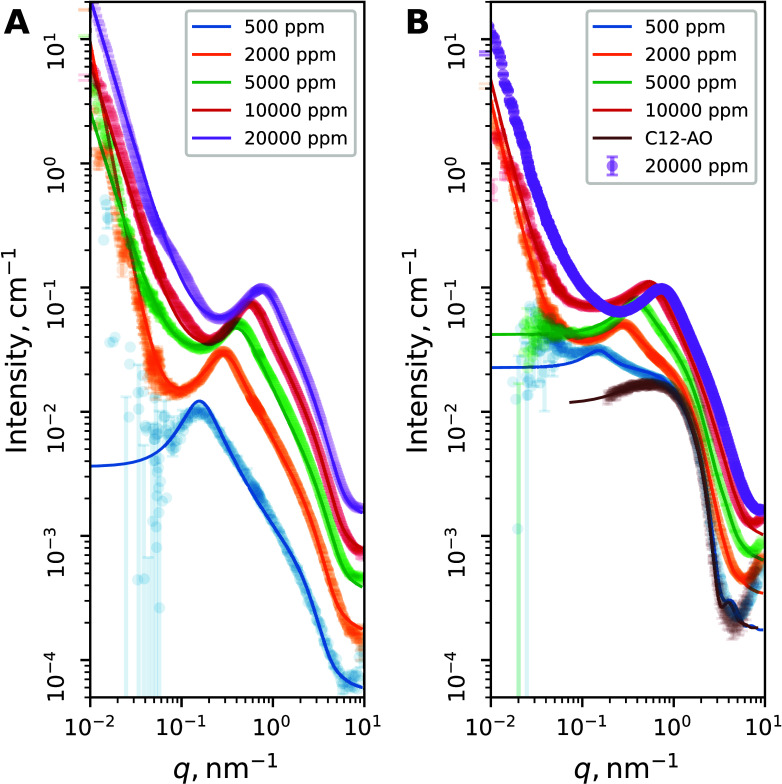
SAXS patterns of (A) the pure polymer solution and (B) solutions containing a polymer and 50 mM C12-AO. Solid lines are fits to the model described in the text. Model details and fit parameters are given in section S6.

The total scattering intensity was calculated as the sum of several contributions. First, scattering from the polyelectrolyte was calculated as shown in eq 2

2 $$I_{p}={\left(\Delta \rho \right)}^{2}\times N\frac{P_{c}\left(q\right)P_{XS\left(q\right)}}{1+\beta P_{c}\left(q\right)c\left(q\right)}$$

where ${\left(\Delta \rho \right)}^{2}$ is ${\left(\rho_{\mathrm{pol}}-\rho_{\mathrm{solvent}}\right)}^{2}$ , *N* is the number density of the polymer, $P_{c}\left(q\right)$ is the form factor of an infinitely thin self-avoiding chain, *P*_XS_ is the cylindrical form factor of the polyelectrolyte cross section, and c(q) is the Fourier transform of the direct chain–chain correlation function, as shown in eq 3.

3 $$c\left(q\right)=\frac{\sin qR}{qR}\times e^{-{\left(q\sigma \right)}^{2}}$$

Secondly, where appropriate, power law *I* ≃ *q* ^–*n*^ was added to account for the presence of the power-law scattering at low *q*, attributed to the large-scale density fluctuations of the polyelectrolyte solution.

Scattering from the micellar solution was modeled as that by monodisperse ellipsoids. High hydration of the surfactant headgroup region leads to the lack of contrast between the solvent and the headgroup. This is in contrast to the SANS measurements, where an elliptical core–shell model was used.^50^ Micelles were found to have a hydrocarbon core of radius *r* = 1.1 ± 0.02 nm and eccentricity ϵ = 1.44 ± 0.01, in accordance with the previous observations by SANS.^51^

Scattering from the mixture of the polymer and surfactant was modeled as a linear combination of scattering from the pure polymer solution and the pure micellar solution of the surfactant. The dimensions of the micelle were allowed to vary; however, only one of the C12-AO samples exhibited slight elongation of the micelle.

Slight differences are observed in the correlation peak intensity and its position, which was shifted toward low *q*. However, the formation of vesicles or other multilayer structures was not detected, contrary to previous observations.^20^ Details of SAXS modeling and tabulated fit parameters are given in section S6.

### Reorganization Mechanism

The analysis of the neutron reflectivity profiles demonstrated that spin-coating of the hydrophilic polymer and amphoteric surfactant produced thin, multilayer microstructured films. The periodicity arises from the alteration between hydrophobic domains (surfactant tails) and hydrophilic domains (polymer and surfactant headgroups). Upon exposure to humidity, the films of the polymer–surfactant complexes reorganize and form a highly ordered multilayer structure with the solvent penetrating hydrophilic regions.

As indicated in Table S1, the Bragg spacing varies between *d* = 32.7 Å for the film dried after the humidity cycle and *d* = 40.3 Å for the swollen film at 80% RH. This is in line with previous observations, reporting a 1.5 nm thickness of the C12-AO monolayer in the binary films of the surfactant and poly­(vinyl alcohol).^52^

Furthermore, the swelling ratio calculated as an increase in the film thickness $a_{d}=\frac{d_{s}-d_{\mathrm{dry}}}{d_{\mathrm{dry}}}=\frac{40.3-32.7}{32.7}=0.23$ corresponds well to the fitted solvent volume fraction ϕ_D_2_O_ in the periodic and uniform parts of the film ϕ_D_2_O_ = 0.22.

The spin-coating process resulted in a relatively dense film, with a density close to that of the bulk components, which is evident from the NR experiments and modeling. This is further confirmed by the solvent penetration in the film at the highest relative humidity, which is only 23% in the hydrophilic regions and close to 0% in the hydrophobic regions. In fact, the solvent is present in the hydrophobic regions only due to the intermixing with neighboring layers. This is in line with the observations from the low-*q* data, where the internal structure of the film can be neglected. The process of the structural transformation is schematically shown in Figure 9.

**Figure 9:**
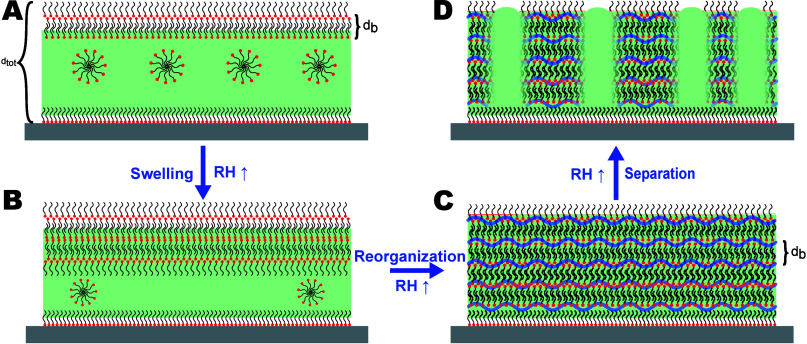
Schematics of structural transformation: (A) As-prepared film; (A low density of micelles is shown for clarity of the schematic representation.) The film at 27% RH (B), 55% RH (C), and 80% RH (D). The polymer matrix is colored solid green; water is colored deep blue. Note the different heights of polymer-rich and surfactant-rich domains.

The neutron reflectivity experiment provided insights into the structure of the dried polymer–surfactant films prepared by spin-coating. It has been found that this process leads to the formation of self-organized multilayer films that undergo reorganization upon exposure to humidity. Furthermore, analysis of the off-specular neutron reflectivity provided valuable estimation of the bending modulus of the multilayer structure at κ = 22*kT* and $\frac{\gamma }{\kappa }=3.6\times 10^{-3}$ . This can be compared with the same ratio for the supported lipid bilayer on the polymer support, $\frac{\kappa }{\gamma }≃10^{-4}$ , when the polymer is swollen and the membrane is flexible and flat and $\frac{\kappa }{\gamma }≃10^{-3}$ when the polymer is collapsed; i.e., the membrane support is more rigid, and the membrane is more distorted.^47^ Therefore, the swollen bilayers formed by the C12-AO surfactant are stiffer than supported lipid bilayers, allowing for the formation of smooth films with excellent mechanical properties.^53^

Moreover, contrast variation experiments have led to the conclusion that in as-prepared films surfactants are uniformly distributed throughout the polymer matrix, and no vertical separation between the polymer and surfactant takes place, contrary to the common phenomena of “blooming” of surfactants in plasticized films, where the surfactant is located almost exclusively on interfaces.^21,22^ In a similar system, in which poly­(vinyl alcohol) (PVA) formed thick films with C12-AO and C14-AO, the surfactants were segregated to the amorphous phase of the polymer in the thick films and exhibited a repeat distance characteristic of a C12-AO bilayer. However, in thin films, surfactants were found to be located primarily on the interfaces of the polymer matrix, i.e., the film–substrate and film–air interface.^52^

In this work, for a similar polymer–surfactant weight ratio, we have found formation of self-organized surfactant multilayers in a polymer matrix upon exposure to humidity. Neutron reflectivity modeling suggests the formation of three to four surfactant bilayers on top of the polymer matrix (cf. Figure 9A), which contains a uniformly dispersed surfactant. Upon exposure to humidity, the film undergoes reorganization, first manifested as buildup of a surface excess of the surfactant on the top interface of the film, as shown in Figure 9B. This is followed by the formation of a surfactant multilayer suspended in the polymer matrix, with the multilayer thickness being equal to the total film thickness (cf. Figure 2C). A further increase in relative humidity leads to lateral separation of the film into surfactant-rich and polymer-rich domains, as shown in Figure 9D. Neutron specular and off-specular reflectivity indicate that the difference in thickness between polymer-rich and surfactant-rich domains is about 3 nm, and an average in-plane size of the polymer-rich domain is 900 nm. It is conceivable that such a transition was due to the change in the wettability of the substrate by the polymer during swelling.

The polymer studied in this work, poly­(methyl vinyl ether-*co*-maleic acid), is highly flexible with a comparatively high *T*_g_ of 154 °C. It is known that physical cross-links formed by the polymer crystals can significantly hinder self-diffusion^54^ as well as diffusion of other molecules. It has been reported that addition of the plasticizer promoted surfactant segregation and diffusion,^21^ which might be attributed to the suppression of the crystallization of the polymer in the case of PVA.^55,56^ Therefore, we suggest that the absence of crystallites allows small molecules to migrate and reorganize in the polymer matrix. It should be noted that a prerequisite for the migration of surfactants is the absence of crystallization of those surfactants. Our GIXD data showed that the formation of surfactant crystals in the polymer matrix was significantly restricted compared to the pure surfactant nanofilm. The surfactant crystals that were formed would undergo humidity-induced melting, allowing for reorganization within the film.

The SAXS pattern from the polymer–surfactant complexes in solution showed characteristics typical of a semidilute polyelectrolyte solution, although with a slight deviation toward chain crossover at higher concentrations.^57^ Such systems are known to have density fluctuations on a length scale much larger than the *R*_g_ of the polymer itself, which results in the appearance of strong low-*q* scattering.^58,59^ SAXS data analysis suggests that the addition of 50 mM C12-AO did not affect the position of the polyelectrolyte correlation peak significantly, whereas low-*q* scattering diminished. This may be indicative of the disruption of the long-range fluctuations and decrease in the polyelectrolyte mesh size, while the short-range correlations due to electrostatic repulsion are preserved. Such long-range fluctuations are challenging to quantify due to the insufficient *q* range of the measurement.

For the solutions with lower polymer concentrations, SAXS data from the mixture of the polymer and surfactant could be described by a linear combination of the scattering from the micellar solution of C12-AO and that of the polymer. The dimensions of surfactant micelles also did not change significantly in comparison to those of the pure micellar solution, indicating a lack of specific interactions between the polymer and surfactant. Accordingly, the structure of the solution can be interpreted as a polyelectrolyte solution, containing surfactant micelles quasi-uniformly dispersed between chains. As mentioned previously, low-*q* scattering was significantly reduced upon addition of the surfactant, suggesting that large-scale density fluctuations were smoothed out in the presence of C12-AO.

This arrangement could lead to the formation of the glassy polymer matrix, which entraps the surfactant micelles during spin-coating. Due to the rapid evaporation of the solvent,^60^ a complete arrangement of surfactants cannot be achieved during spin-coating. With an increase in relative humidity, the increase in the partial pressure of water vapor results in the penetration of the vapor inside the polymer and swelling of the film, which drives migration of the surfactant molecules and formation of the multilayer structures due to self-organization of the surfactants.

Multilayer structures incorporating antimicrobial surfactants have been shown to display a higher antimicrobial efficiency, as the diffusion of small molecules is favored, compared to the strong binding of surfactants to the polymer.^13^ This study thus demonstrates that spin-coating provides a facile method of producing a polymer–surfactant multilayer film with a high surfactant content, which is beneficial for the development of antimicrobial films with prolonged efficacy longevity.

## Conclusion

In this study, we have investigated the structure of a nanofilm formed by complexation between an amphoteric amine oxide surfactant and an anionic polyelectrolyte under conditions under which the Coulombic interaction is suppressed. The solution structure of the polymer–surfactant complexes was also studied and correlated to the nanofilm structure.

The neutron reflectivity experiment revealed the formation of layered structures in the porous spin-coated thin film and its subsequent reorganization into multilayers of the surfactant suspended in the glassy polymer matrix upon exposure to humidity. X-ray scattering revealed the presence of the multiscale organization of the film, which enables complex structural reorganization. Contrary to expectations, multilayer structures were not present in solution, as revealed by SAXS, which showed the presence of small ellipsoidal micelle-like aggregates between polyelectrolyte chains in the semidilute regime. Modeling of the off-specular neutron scattering and GISAXS data revealed the presence of shallow meso- and macropores on the surface of the film. The formation of pores is crucial for swelling of the polymer matrix, enabling migration of surfactants and their subsequent reorganization into multilayer structures. This reorganization is further enabled by the humidity-induced melting of surfactant nanocrystals embedded in the film. This process would allow the antimicrobial surfactants to be released upon exposure to humidity, further prolonging the disinfecting activity of the self-assembled film.

## Supplementary Material



## Notes

Details of the characterization of the P–S films using NR, GID, XRR, and GISAXS; details of the analysis of solution SAXS; and reciprocal space maps obtained from off-specular NR (PDF)
