# Supplementary material for: Humidity-Induced Structural Transformation in Self-Organized Polymer–Surfactant Multilayer Nanofilms
Source: Langmuir. 2025 Oct 23;41(43):29335–45. doi: 10.1021/acs.langmuir.5c04237 (PMC12593366; doi:10.1021/acs.langmuir.5c04237)
Supplement: Supplementary file 1 [file la5c04237_si_001.pdf]

Supporting Information: Humidity induced structural transformation in self-organised polymer-surfactant multilayer nanofilms

Egor A. Bersenev[a,b,*], Phillip Gutfreund[c], Valentina Rein[a], Andrei P. Chumakov[d], Oleg V. Konovalov[a,**], Wuge H. Briscoe[b,1]

[a]*European Synchrotron Radiation Facility (ESRF), 71 avenue des Martyrs, Grenoble, 38000, France*
[b]*School of Chemistry, University of Bristol, Cantock's Close, Bristol, BS8 1TS, United Kingdom*
[c]*Institut Laue–Langevin (ILL), 71 Avenue des Martyrs, Grenoble, 38000, France*
[d]*Deutsches Elektronen-Synchrotron, Notkestrasse 85, Hamburg, 22607, Germany*

Keywords: polymer-surfactant interaction, thin film, neutron reflectometry

[*]egor.bersenev@bristol.ac.uk
[**]konovalo@esrf.fr
[1]wuge.briscoe@bristol.ac.uk

1. Supplementary Materials: Humidity induced structural transformation in self-organised polymer-surfactant multilayer nanofilms

S1. Low-q fit of NR to the uniform film model

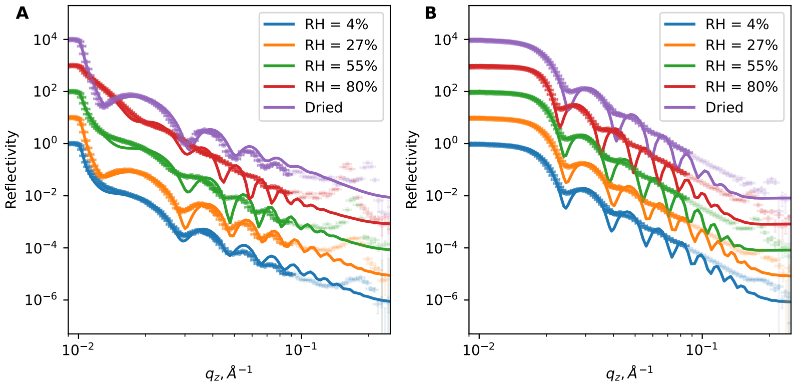

Figure S1: Neutron reflectometry profiles. A. Film, containing h-surfactant; B. Films, containing d-surfactant. Thin solid lines represent fits to the uniform fit model. Curves are shifted two orders of magnitude for visibility. Colours and order are the same as in the main text.

S2. Fit of the Bragg peak in the NR curves

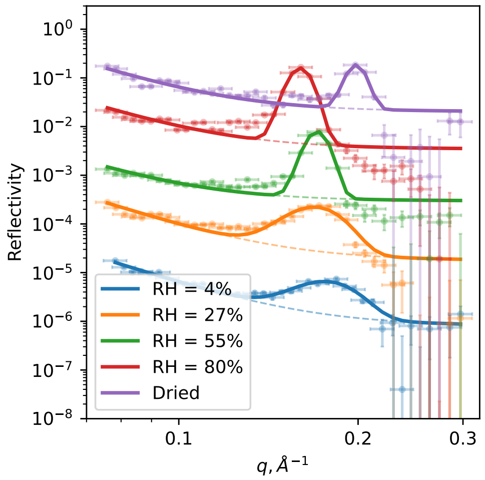

Figure S2: Neutron reflectometry profiles of the films, containing h-surfactant. Thin solid lines represent fits to the Gaussian peak with a q^{-4} background. Curves are shifted two orders of magnitude for visibility.

S3. Macroscopic parameters of the P-S films

RH	d_{tot}, nm	σ_{tot}, nm	q_{B}, Å^{-1}	w_{B}, Å^{-1}	τ, nm	N_{L}	N_L^{max}	$\phi_{\mathrm{D_2O}}$, %
0	34.7	0.80	0.177	0.054	13.4 ± 1.4	3.8 ± 0.4	9.7 ± 0.2	0
27	34	0.83	0.169	0.036	15.5 ± 1.4	4.2 ± 0.4	9.2 ± 0.2	4
55	36	1.57	0.171	0.017	37.5 ± 1.6	10.2 ± 0.4	9.8 ± 0.4	17
80	40	2.02	0.160	0.015	38.8 ± 1.6	10 ± 0.4	10.3 ± 0.5	27
5	33	1.97	0.199	0.018	37.9 ± 4.7	12 ± 1.5	10.3 ± 0.5	5

Table S1: Macroscopic parameters extracted from the NR profiles. Non-indicated errors are less than the last significant digit.

S4. X-ray reflectivity

Film of pure C12-AO surfactant was characterized using X-ray reflectivity. Film was prepared by spin-coating a 50 mM solution of C12-AO at 4000 rpm on a cleaned Si wafer. Reflectivity profile was fitted to a multilayer model with a wetting layer towards the substrate. 3 slabs were used to model a repeat unit. Model incorporated porosity of the film to account for packing defects. It was found to be ϕ_{air} = 8.2 ± 0.5%.

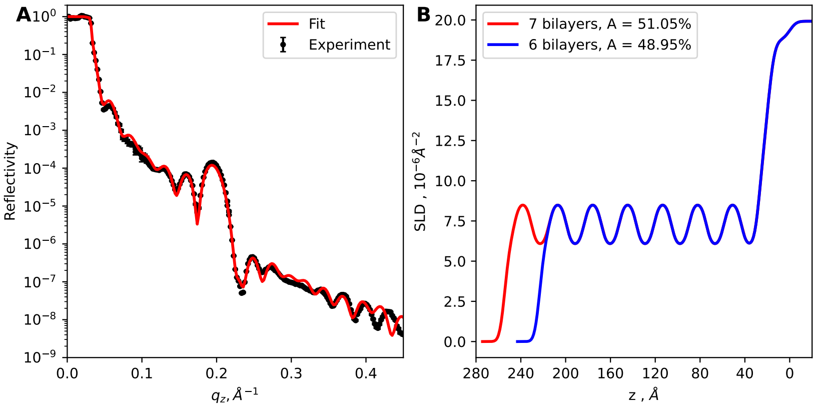

Figure S3: X-ray reflectivity of C12-AO film, coated at 4000 rpm from 50 mM solution. A. XRR profile and fit to the 3-slab multilayer model. B. SLD profiles, obtained from the fitting.

S5. Grazing-incidence X-ray Diffraction

Peak	q_{xy} Å^{-1}	σ Å^{-1}	I_p
C12-AO film, peak 1	1.28	0.022	69520 ± 1041
C12-AO film, peak 2	1.43	0.016	202399 ± 1201
P-S film, peak 1	1.27	0.033	13454 ± 555
P-S film, peak 2	1.43	0.022	27163 ± 548

Table S2: Fit parameters of the Bragg peaks, integrated in q_z direction. Fits are shown in the Figure S5

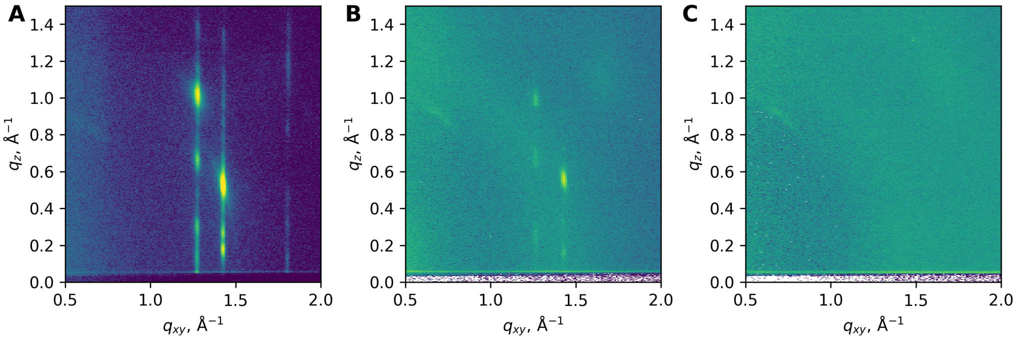

Figure S4: Reciprocal space maps, reconstructed from the scanning GID experiment. A. Film of C12-AO. B. Film of P-S complex, as-prepared, RH=0%. C. Film of P-S complex, RH=60%.

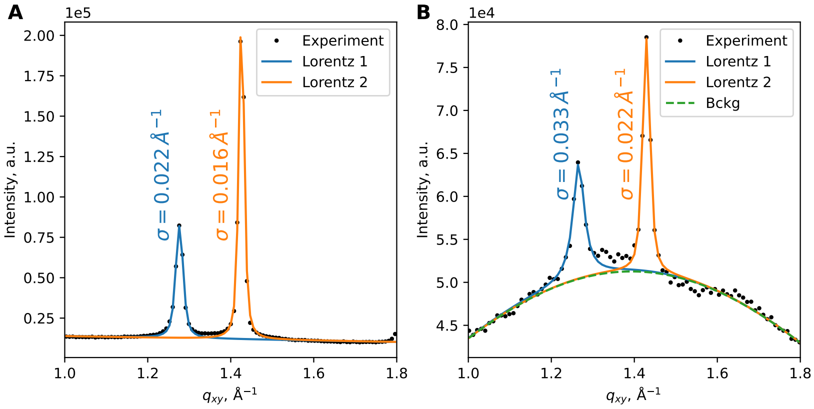

Figure S5: q_z-integrated GID profiles. A. Film of C12-AO. B. Film of P-S complex, as-prepared, RH=0%. Solid lines are fits to the Lorentzian peak model with linear background in A and Gaussian shape for amorphous scattering in B. Parameters are given in the Table S2.

Peak	q_z, Å^{-1}	σ, Å^{-1}	I_p
C12-AO film	0.53	0.050	2263 ± 5
P-S film	0.56	0.059	233 ± 2

Table S3: Fit parameters of the most intense Bragg peak in q_z direction. Fits are shown in the Figure S6

S6. SAXS analysis

Scattering from the polyelectrolyte solution was analysed using the Pedersen-Shturtenberg approach [1], using the form-factor in the equation S1 for self-avoiding chains with excluded volume:

$$S(q, L, b) = S_{exv}(q, L, b) + C\left(\frac{L}{b}\right)\left[\frac{4}{15} + \frac{7}{15u} - \left(\frac{11}{15} + \frac{7}{15u}\right) \cdot e^{-u}\right] \cdot \frac{b}{L} \qquad \text{(S1)}$$

where $S_{exv}(q, L, b)$ is given by an equation 13 from [1] and $C\left(\frac{L}{b}\right) = a_4/\left(L/b\right)^{p_3}$ with $a_4 =$

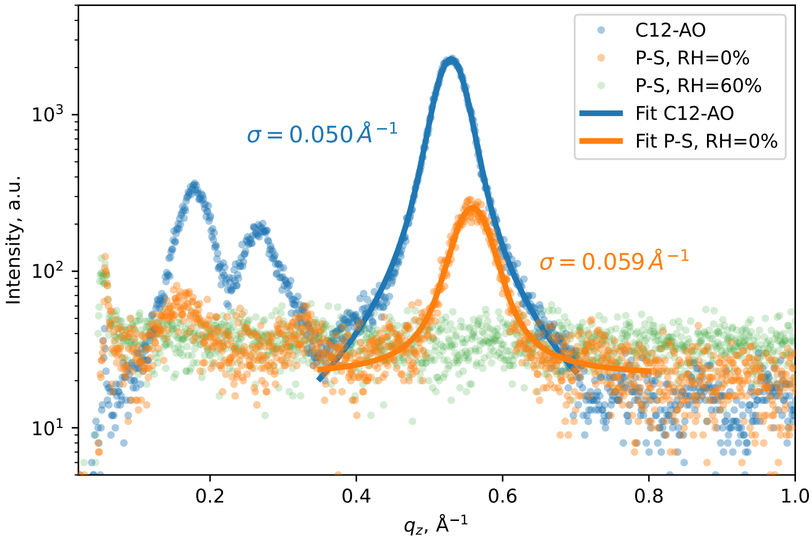

Figure S6: q_z cuts of Bragg rods, located at $q_{xy} = 1.43\,\text{Å}^{-1}$ from images in the Figure S4. Solid lines are fits to the Voigt peak model with linear background. Parameters are given in the Table S3.

3.06 and $p_3 = 0.44$. Power law was parametrized as $I = c_2 \cdot q^{c_1}$.

Parameter	500 ppm	2000 ppm	5000 ppm	10000 ppm	20000 ppm
L, nm	320	320	320	320	320
b_k, nm	14.6 ± 0.7	6.9 ± 0.1	6.26 ± 0.06	4.59 ± 0.04	3.45 ± 0.01
σ, nm	10.98 ± 0.25	5.1 ± 0.1	3.64 ± 0.04	2.71 ± 0.02	1.70 ± 0.01
R_{xs}, nm	1.22 ± 0.06	0.76 ± 0.01	0.74 ± 0.01	0.64 ± 0.01	0.58 ± 0.01
β	48.9 ± 2.7	64.3 ± 2.1	91 ± 3	186.1 ± 3.5	191 ± 4
$c_{pol} \cdot 10^{-8}$	1.85 ± 0.02	8.5 ± 0.03	21.4 ± 0.1	48.4 ± 0.2	89 ± 1
$I_b \cdot 10^{-4}$, cm^{-1}	0.74 ± 0.11	1.6 ± 0.05	4.1 ± 0.1	6.96 ± 0.1	13.6 ± 0.2
c_1	-	−3.88 ± 0.03	−2.18 ± 0.05	−2.36 ± 0.03	−2.45 ± 0.01
c_2	-	$(1.6 \pm 0.2) \cdot 10^{-7}$	$(9.5 \pm 1.5) \cdot 10^{-5}$	$1.1 \cdot 10^{-4}$	$2.6 \cdot 10^{-4}$

Table S4: Fit parameters for pure polymer solutions

Parameter	500 ppm	2000 ppm	5000 ppm	10000 ppm
L, nm	320	320	320	320
b_k, nm	14.5	12 ± 3	3.5 ± 0.3	3.7 ± 0.9
σ, nm	11	6.1 ± 0.2	3.41 ± 0.06	2.46 ± 0.15
R_{xs}, nm	1.14 ± 0.7	1 ± 0.1	0.78 ± 0.02	0.6 ± 0.06
β	16 ± 4	79 ± 4.5	90 ± 2	193.6 ± 62.3
$c_{pol} \cdot 10^{-8}$	1.42 ± 0.02	11 ± 2	25 ± 1	45 ± 7
$I_b \cdot 10^{-4}$, cm^{-1}	1.7 ± 0.6	3.2 ± 0.2	7.1 ± 2.5	9.4 ± 0.4
c_1	-	-3.1 ± 0.1	-	-3 ± 0.3
c_2	-	$(2.4 \pm 0.7) \cdot 10^{-6}$	-	$(4.9 \pm 0.6) \cdot 10^{-6}$
R_a, nm	1.78 ± 0.08	1.62 ± 0.02	1.45 ± 0.4	4.8 ± 0.8
R_b, nm	1.2 ± 0.07	1.2 ± 0.1	0.85 ± 0.01	1 ± 0.1
c_s, mM	50	50	50	50
c_f, mM	9.8 ± 4.3	22 ± 18	7 ± 2	0.5 ± 0.2

Table S5: Fit parameters for P+S solutions, containing 50 mM of C12-AO

Scattering from micellar solution was modelled as monodisperse ellipsoids of revolution, corresponding to the hydrocarbon core of the micelle. Constraints were put on the scattering length density of the core density and number density of the micelles in solution. Rescaled Hayter-Penfold structure factor was used to model repulsion between micelles, with radius taken as an average between half-axes R_a and R_b. Charge was calculated as $q = \alpha \cdot N_{agg}$.

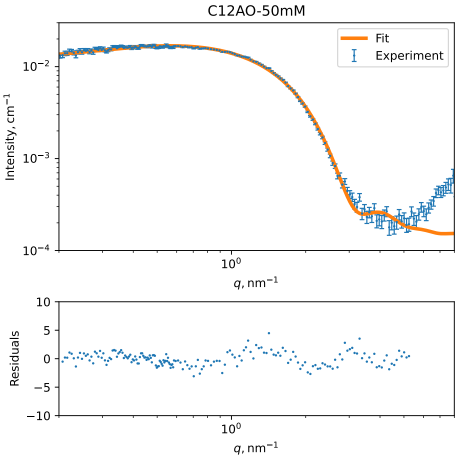

Figure S7: SAXS pattern of 50 mM solution of C12-AO. Solid line corresponds to the fit to the ellipsoid model with $\chi^2 = 1.45$.

Parameter	R_a, nm	R_b, nm	c_f, mM	I_b, cm^{-1}	α, %
Value	1.07 ± 0.02	1.58 ± 0.01	9.75 ± 0.25	$(1.45 \pm 0.09) \cdot 10^{-4}$	7.2 ± 0.1

Table S6: Fit parameters for micellar solution of C12-AO

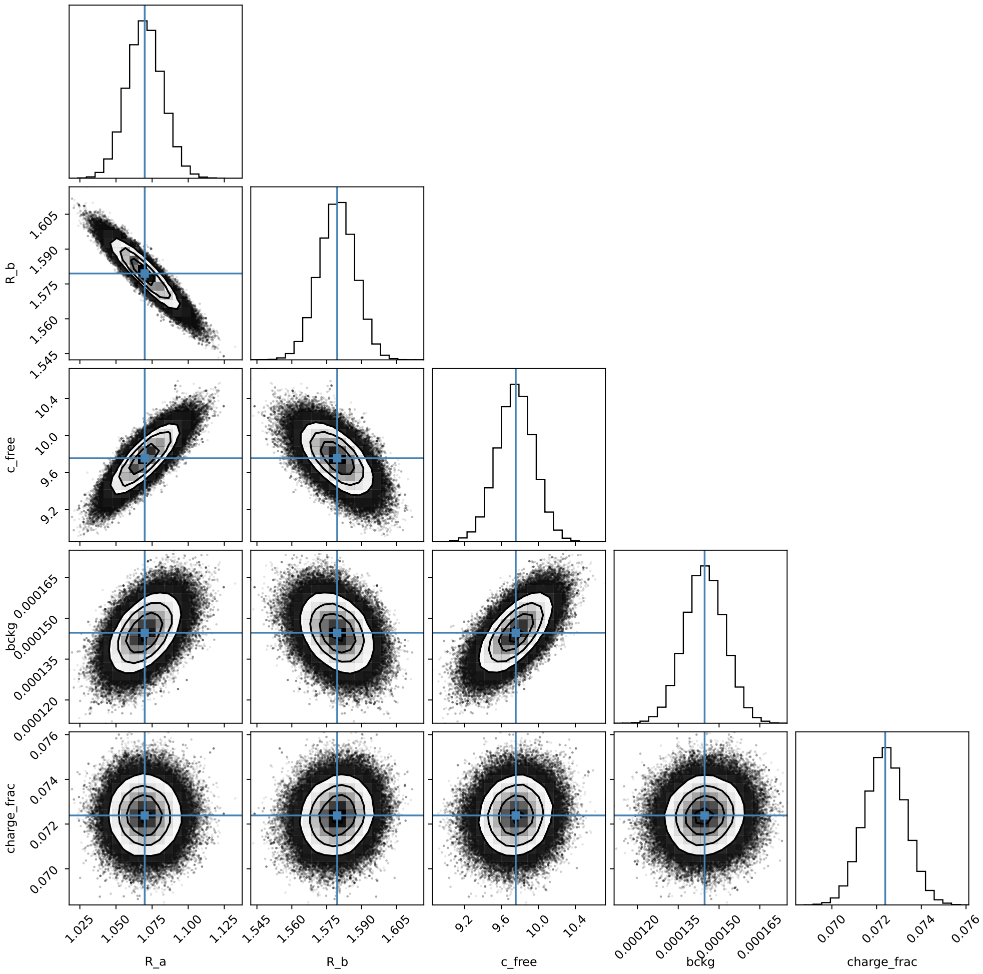

Figure S8: Posterior distributions of the model parameters for the pure 50 mM C12-AO solution. Thin blue lines indicate values, obtained from the differential evolution fit and used as a centre of prior distribution.

S7. GISAXS experiments and simulations

GISAXS simulations were conducted using BornAgain code and custom python scripts. Model was constructed as follows: Si substrate, thin SiO2 layer and uniform polymer layer with polydisperse semi-ellipsoidal pores in the film surface. Semi-axes of the ellipsoid in the film plane were a and b, depth c. Area,covered by the particles was calculated as $a \cdot b \cdot n_p$, where n_p is the number density of scatterers, expressed in nm^{-2}. Film thickness was set to 40 nm, and SLD to $\mathbf{SLD}_\mathrm{f} = 8.3 \cdot 10^{-4}\,\mathrm{nm}^{-2}$ for RH = 11% and RH = 25%, and $\mathbf{SLD}_\mathrm{f} = 10 \cdot 10^{-4}\,\mathrm{nm}^{-2}$ for other humidities. GISAXS signal from the film at RH = 78% was calculated as scattering on correlated roughness of $\sigma_{tot} = 1\,\mathrm{nm}$ with Hurst parameter $H = 0.5$ and correlation length $\xi = 200\,\mathrm{nm}$. Evolution of pore dimensions and area, covered by pores in shown in the Figure S9.

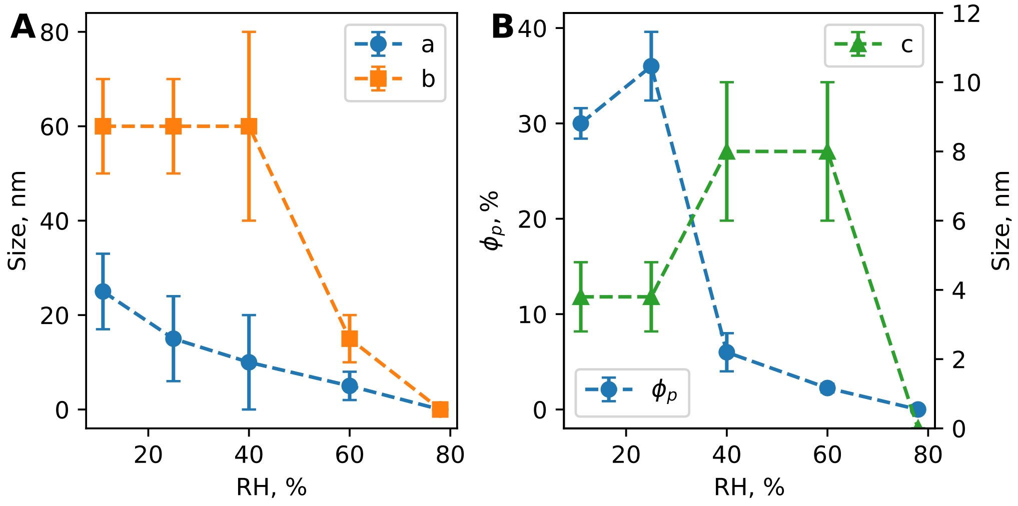

Figure S9: Simulation parameters of GISAXS images. A. a and b parameters of the ellipsoids. B. Share of the surface, covered in pores ϕ_p and depth of the ellipsoid c.

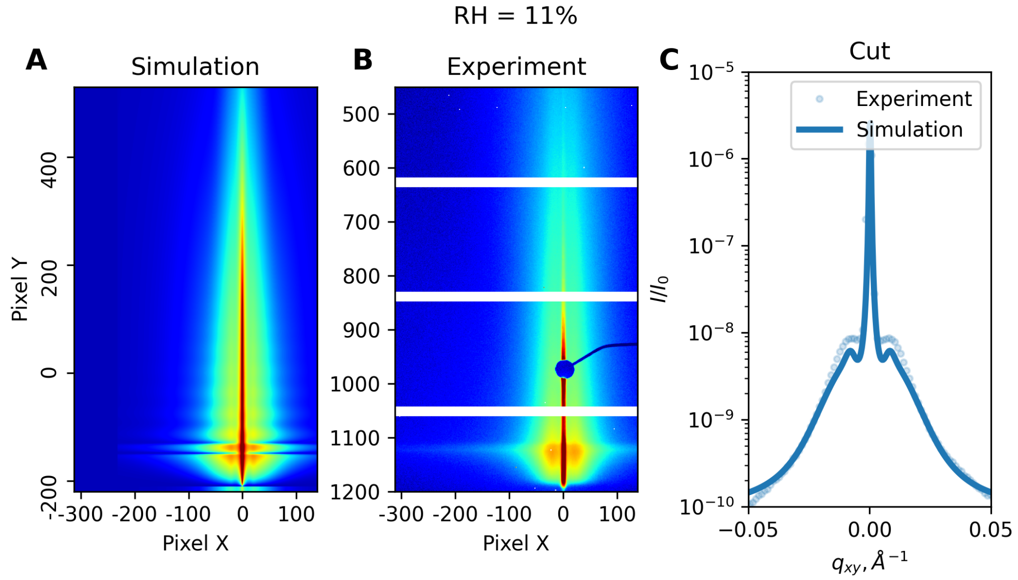

Figure S10: GISAXS detector image, RH=11%. A: Simulated image, B: Experimental data, C: q_{xy} cuts from experimental and simulated image.

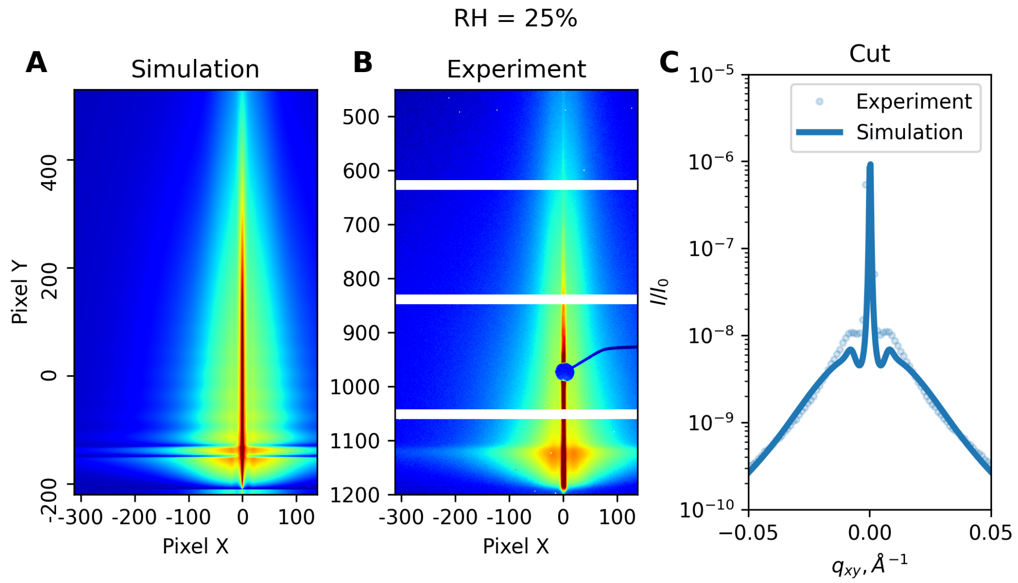

Figure S11: GISAXS detector image, RH=25%. A: Simulated image, B: Experimental data, C: q_{xy} cuts from experimental and simulated image.

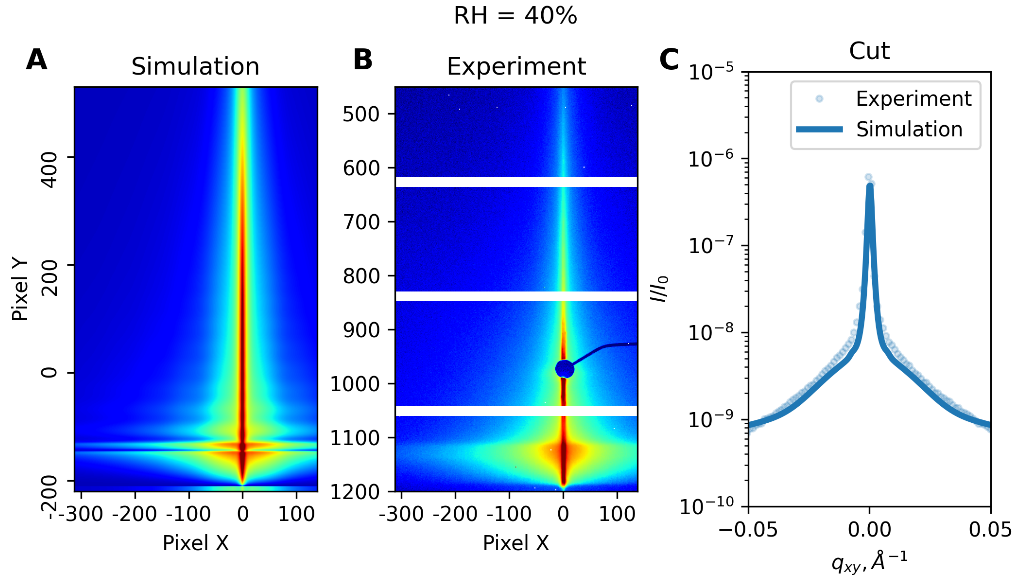

Figure S12: GISAXS detector image, RH=40%. A: Simulated image, B: Experimental data, C: q_{xy} cuts from experimental and simulated image.

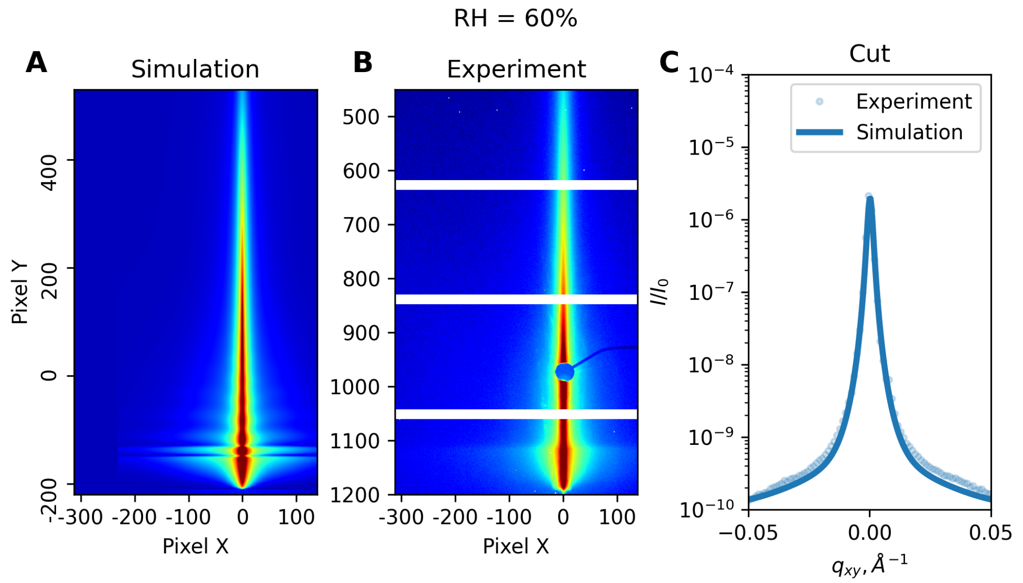

Figure S13: GISAXS detector image, RH=60%. A: Simulated image, B: Experimental data, C: q_{xy} cuts from experimental and simulated image.

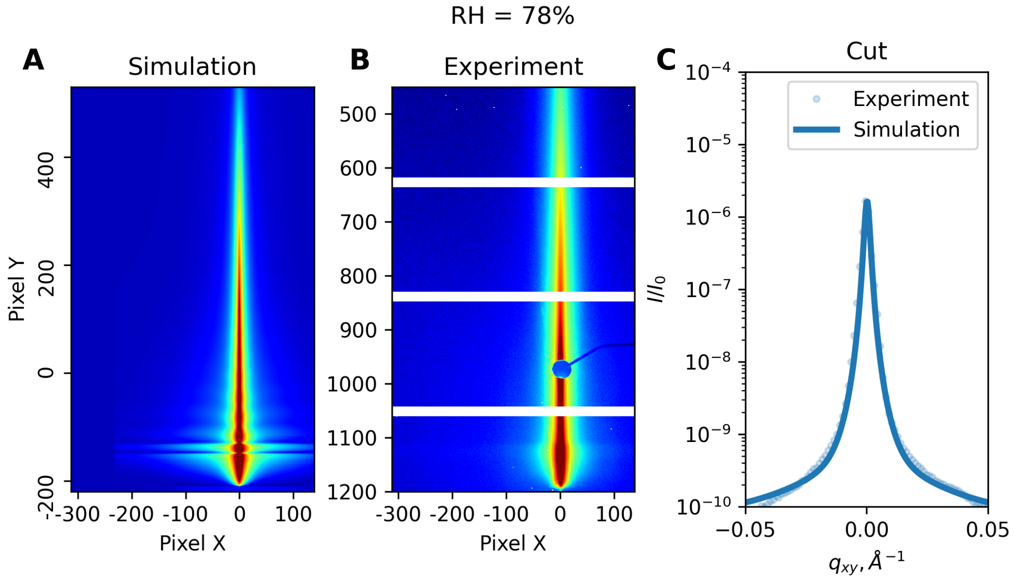

Figure S14: GISAXS detector image, RH=78%. A: Simulated image, B: Experimental data, C: q_{xy} cuts from experimental and simulated image.

S8. Off-specular neutron reflectivity at high-q

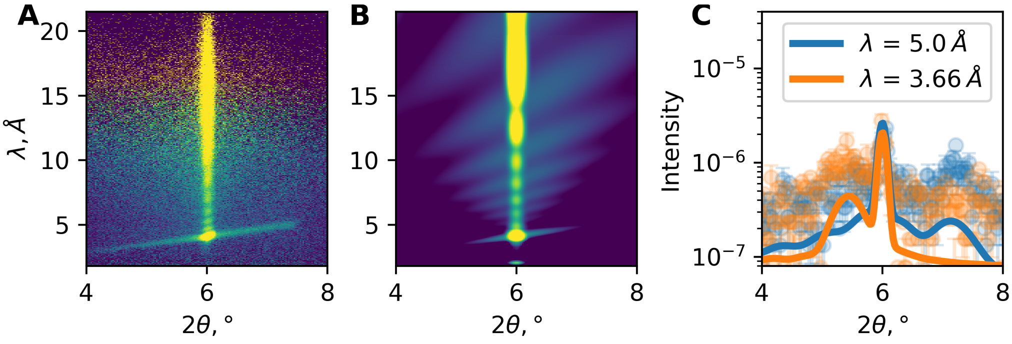

Figure S15: Off-specular neutron reflectivity at high-q, RH=80%. A: h-contrast, experiment, B: h-contrast, calculated intensity according to the model described in the text, C: h-contrast, cuts at $\lambda = 5$ Å and $\lambda = 3.5$ Å

S9. Off-specular neutron reflectivity at low-q

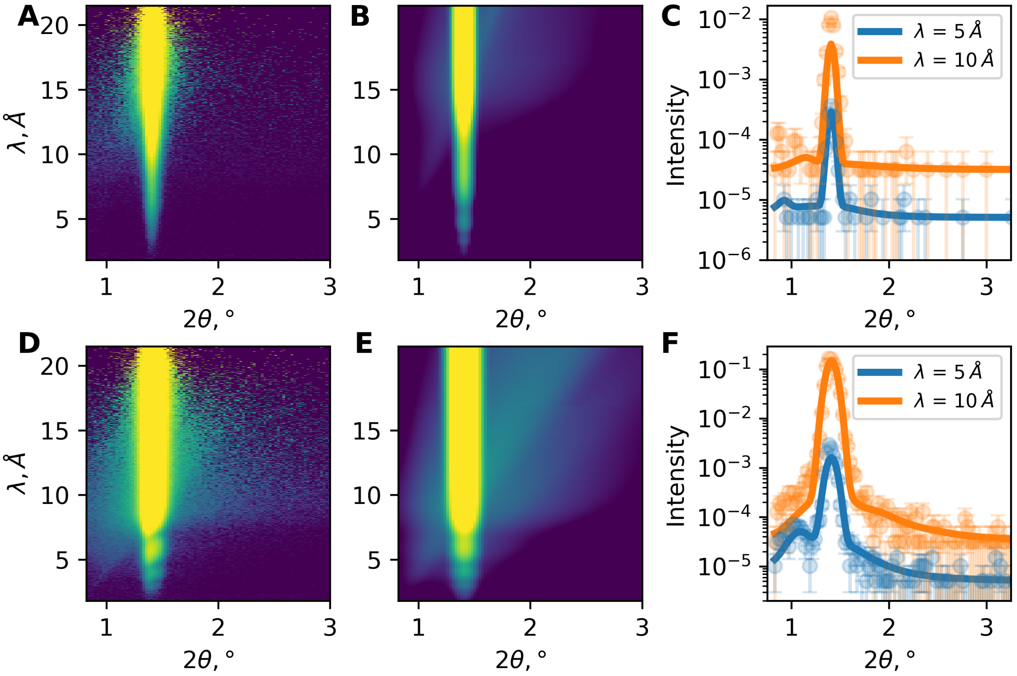

Figure S16: Off-specular neutron reflectivity, RH=80%. A: h-contrast, experiment, B: h-contrast, calculated intensity according to the model described in the text, C: h-contrast, cuts at $\lambda = 5$ Åand $\lambda = 10$ Å,D: d-contrast, experiment, E: d-contrast, calculated intensity according to the model described in the text, F: d-contrast, cuts at $\lambda = 5$ Åand $\lambda = 10$ Å

S10. Off-specular neutron reflectivity at RH=5%

Off-specular scattering was modelled as monodisperse cylinders with $R = 200 \pm 50$ nm, surface coverage $\phi = 9$ % and depth $c = 3$ nm.

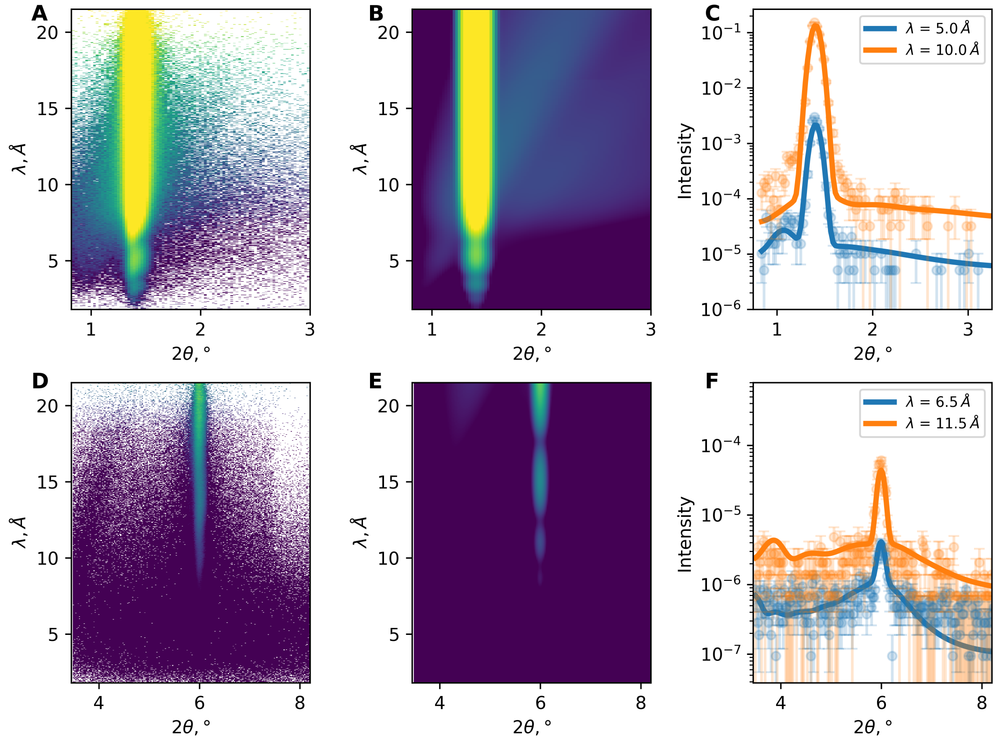

Figure S17: Off-specular neutron reflectivity, RH=5%. A: d-contrast, experiment, low-q, B: d-contrast, calculated intensity according to the model described in the text, low-q, C: d-contrast, cuts at $\lambda = 5$ Å and $\lambda = 10$ Å,D: d-contrast, experiment, high-q, E: d-contrast, calculated intensity according to the model described in the text, high-q, F: d-contrast, cuts at $\lambda = 6.5$ Å and $\lambda = 11.5$ Å

References

[1] J. S. Pedersen, P. Schurtenberger, Scattering Functions of Semiflexible Polymers with and without Excluded Volume Effects, Macromolecules 29 (23) (1996) 7602–7612, publisher: American Chemical Society. doi:10.1021/ma9607630.
URL https://doi.org/10.1021/ma9607630
